# Impaired synaptic function and hyperexcitability of the pyramidal neurons in the prefrontal cortex of autism-associated *Shank3* mutant dogs

**DOI:** 10.1186/s13229-024-00587-4

**Published:** 2024-01-31

**Authors:** Feipeng Zhu, Qi Shi, Yong-hui Jiang, Yong Q. Zhang, Hui Zhao

**Affiliations:** 1 State Key Laboratory for Molecular Developmental Biology, Institute of Genetics and Developmental Biology, Chinese Academy of Sciences, Beijing, 100101 China; 2 College of Life Sciences, University of Chinese Academy of Sciences, Beijing, 100049 China; 3 Department of Genetics and Neuroscience, Yale University School of Medicine, New Haven, CT 06510 USA; 4 School of Life Sciences, Hubei University, Wuhan, 430415 China; 5 Department of Neurobiology, School of Basic Medical Sciences, Capital Medical University, Beijing, 100069 China

**Keywords:** Shank3, Autism spectrum disorder, Synaptic transmission, Excitability, Dog

## Abstract

**Background:**

*SHANK3* gene is a highly replicated causative gene for autism spectrum disorder and has been well characterized in multiple *Shank3* mutant rodent models. When compared to rodents, domestic dogs are excellent animal models in which to study social cognition as they closely interact with humans and exhibit similar social behaviors. Using CRISPR/Cas9 editing, we recently generated a dog model carrying *Shank3* mutations, which displayed a spectrum of autism-like behaviors, such as social impairment and heightened anxiety. However, the neural mechanism underlying these abnormal behaviors remains to be identified.

**Methods:**

We used *Shank3* mutant dog models to examine possible relationships between *Shank3* mutations and neuronal dysfunction. We studied electrophysiological properties and the synaptic transmission of pyramidal neurons from acute brain slices of the prefrontal cortex (PFC). We also examined dendrite elaboration and dendritic spine morphology in the PFC using biocytin staining and Golgi staining. We analyzed the postsynaptic density using electron microscopy.

**Results:**

We established a protocol for the electrophysiological recording of canine brain slices and revealed that excitatory synaptic transmission onto PFC layer 2/3 pyramidal neurons in *Shank3* heterozygote dogs was impaired, and this was accompanied by reduced dendrite complexity and spine density when compared to wild-type dogs. Postsynaptic density structures were also impaired in *Shank3* mutants; however, pyramidal neurons exhibited hyperexcitability.

**Limitations:**

Causal links between impaired PFC pyramidal neuron function and behavioral alterations remain unclear. Further experiments such as manipulating PFC neuronal activity or restoring synaptic transmission in *Shank3* mutant dogs are required to assess PFC roles in altered social behaviors.

**Conclusions:**

Our study demonstrated the feasibility of using canine brain slices as a model system to study neuronal circuitry and disease. *Shank3* haploinsufficiency causes morphological and functional abnormalities in PFC pyramidal neurons, supporting the notion that *Shank3* mutant dogs are new and valid animal models for autism research.

**Supplementary Information:**

The online version contains supplementary material available at 10.1186/s13229-024-00587-4.

## Background

SH3 and multiple ankyrin repeat domain (SHANK) family genes encode the scaffolding proteins SHANK1–3 at postsynaptic densities (PSD) in excitatory synapses, with *SHANK* gene mutations associated with autism spectrum disorder (ASD) [1]. *Shank3* deficiency has been extensively studied in animal models, especially in mice, but also in rats and nonhuman primates [2]. Previous rodent studies have shown that *Shank3* deletion caused abnormal behaviors relevant to ASD, including social interaction deficits and repetitive behaviors [3–5], which provide evidence supporting the face validity of *Shank3* deficiency mouse as an autism model. Importantly, altered synaptic development, impaired synaptic function, and reduced dendritic spines at specific brain regions, due to *Shank3* loss, are hypothesized as the neural substrates underlying these behavioral impairments [6–8]. However, the translation of *Shank3* mutant rodent model outcomes to ASD patients is somewhat limited as brain structure and behavior differences exist between humans and rodents. Therefore, new animal models are required to further study autism mechanisms and treatments.

Growing evidence now suggests that the domestic dog can be used as an effective animal model to investigate human social behaviors and mental disorders as they have developed sophisticated cross-species emotional and social processing abilities during their long coevolutionary history with humans [9–11]. Additionally, dogs harbor brain sulcus and gyrus structures that resemble those in humans [12]. More importantly, dogs are more readily available than monkeys as they reproduce more quickly (1 versus 5 years to adulthood) and are more prolific (one versus multiple offspring per birth). We recently reported that *Shank3* mutant dogs, generated by CRISPR/Cas9 editing, exhibited ASD-like behaviors, such as social withdrawal, elevated anxiety, and hypersensitive to deviant tones [13, 14], indicating that these dogs were effective ASD animal models. However, defining neuronal activity and synaptic function in such animals is required to understand the causal relationships between neuronal dysfunction and abnormal social behaviors caused by *Shank3* mutations.

The prefrontal cortex (PFC) has essential roles in cognitive control, coordinating memory, and executive activity [15]. Previous patient and animal model studies implicated PFC as an important structure in social functions [2, 16, 17] as patients with ASD have structural and functional abnormalities in the PFC [18, 19]. The chemogenetic activation of pyramidal neurons in the PFC of *Shank3* mutant mice elevated glutamatergic synaptic function in the PFC and restored social preference behaviors [20]. Given the crucial role of the PFC in regulating such social behaviors, we sought to characterize possible defects in PFC neuronal structure and function in *Shank3* mutant dogs.

Electrophysiological recording in acute brain slices is a unique technique for studying neuronal activity and synaptic transmission due to its millisecond resolution and nanomolar sensitivity [21, 22]. Because neural network connections are preserved and structural alterations are minimal when compared to organotypic cultures for a few days, studies using acute brain slices from mammalian brains have been widely conducted [21]. Here, we first established physiological recording protocols in fresh brain slices from dogs to investigate the electrophysiological characteristics of single PFC neurons. We then examined dendritic and synaptic alterations in pyramidal neurons caused by *Shank3* mutations. We observed that *Shank3* loss led to hyperexcitable neuronal activity and a deficiency in glutamatergic synaptic transmission in the PFC of *Shank3* mutant dogs. To our knowledge, this is the first report investigating individual neuronal properties in an autism-associated dog model. Our findings provided a possible neurobiological explanation for abnormal social behaviors in *Shank3* mutant dogs.

## Materials and methods

### Animals

Six Beagle dogs aged ranging from 3 to 4 months were used for all experiments in this study (Additional file 1: Table S1). Among them, three male wild-type Beagle dogs were from Sinogene Ltd (Beijing, China). Three *Shank3* mutant dogs (one female and two male) were heterozygous for a 496 bp deletion in exon 21 of the *Shank3* gene (-496 bp/ + ; + after the slash denotes a wild-type copy of the gene); this mutation generated a frameshift and a truncated protein disrupting the proline-rich domain of Shank3 [12]. The mutant dogs showed a reduced Shank3 protein level in the brain and displayed autism-like social deficits, including social withdrawal, elevated anxiety, and reduced social interactions with humans [13]. Each dog was housed in a separate cage (1 × 1 × 1 m^3^) and maintained on a 12 h/12 h dark–light cycle (light on: 07:00–19:00), with a humidity of 40–60% and a temperature of 22–24 °C. The dogs were fed with regular canine food (Keao Xieli Feed Co.Ltd, Beijing, China) twice daily at 08:00–10:00 and 15:30–17:00. All animal experiments and protocols were approved in advance by The Animal Care and Use Committee of the Institute of Genetics and Developmental Biology (AP2023024).

### Surgery and brain slice preparation

The dogs were anesthetized by simultaneous administration of dexmedetomidine hydrochloride (25 μg/kg, intramuscular), Zoletil 50 (5 mg/kg, intramuscular), and propofol (5 mg/kg, intravenous). After deep anesthesia, the dogs were sacrificed and transcardially perfused with ice-cold 0.9% saline (Kexing, China). The whole brain was removed, placed in a large beaker and kept submerged in cold (4 °C) cutting solution containing (in mM): 110 C_5_H_14_NClO, 2.5 KCl, 25 NaHCO_3_, 1.3 NaH_2_PO_4_, 7 MgCl_2_, 0.5 CaCl_2_, and 25 glucose (adjusted to pH 7.3–7.4, oxygenated with 95% O_2_ and 5% CO_2_). The PFC was sampled between the anterior and middle third of the medial frontal gyrus according to The Beagle Brain in Stereotaxic Coordinates [23]. A small block of tissue containing the PFC was excised and immediately transported to the laboratory. Coronal brain slices (300 μm thickness) were cut using a vibratome (Leica VT1200s, Wetzlar, Germany). The PFC slices were incubated for 30 min at 37 °C and then maintained at room temperature (22–25 °C) in the artificial cerebrospinal fluid (aCSF) containing (in mM): 125 NaCl, 2.5 KCl, 25 NaHCO_3_, 1.3 NaH_2_PO_4_, 1.3 MgCl_2_, 2.5 CaCl_2_, and 25 glucose (adjusted to pH 7.3–7.4, oxygenated with 95% O_2_ and 5% CO_2_) for physiological recording.

### Electrophysiological recording

The brain slice was transferred to a recording chamber and constantly perfused with oxygenated aCSF at 25 °C at a rate of 1.5–2.0 ml/min. Cells were visualized using IR-DIC optics on an inverted Olympus BX51WI microscope. Layer 2/3 pyramidal neurons were identified by their morphology. Whole-cell recordings were performed with Multiclamp 700B amplifier and Digidata Digitizer 1550B (Molecular Devices Corporation, Sunnyvale, CA, USA). The signals were acquired at 10 kHz and filtered at 2 kHz. Cells were voltage-clamped at − 70 mV with 4–6 MΩ patch pipettes. The access series resistance of the neurons used for analysis was < 25 MΩ.

For neuronal activity recordings, the pipette solution contained (in mM): 130 K-gluconate, 10 HEPES, 5 KCl, 4 MgCl_2_, 0.2 EGTA, 4 MgATP, 0.3 NaGTP, 10 Na_2_-Phosphocreatine. Biocytin (2 mg/ml, B4261, Sigma) was also added to the pipette solution to allow for post hoc identification of the location and morphology of recorded neurons. The intrinsic and active membrane properties were generally recorded in the presence of CNQX (6-cyano-7-nitroquinoxaline-2,3-dione; 10 μM), AP5 (2-Amino-5-phosphonopentanoic acid; 50 μM), and PTX (50 μM). Resting membrane potential was recorded immediately after breaking the seal. For analyzing action potential (AP) properties, the following parameters were calculated: AP threshold (the membrane potential of the first time dVm/dt reaching 2 mV/ms), AP amplitude (the subtraction between spike peak and AP threshold), half-width (the duration between membrane potential shoot over and drop below half amplitude), AP rise time (time between 10 and 90% of spike amplitude on depolarizing phase), decay time (time between 10 and 90% of spike amplitude on repolarizing phase), after hyperpolarization amplitude (membrane potential difference between AP threshold and the lowest potential in the hyperpolarization phase), and AHP latency (the time from AP threshold to the lowest potential of spike).

For synaptic transmission recordings, the pipette solution contained (in mM): 135 CsMeSO_3_, 10 HEPES, 1 EGTA, 4 MgATP, 0.3 NaGTP, 8 Na_2_-Phosphocreatine, 3.3 QX314 bromide (adjusted to pH 7.3 with CsOH). Evoked excitatory postsynaptic currents (EPSCs) and inhibitory postsynaptic currents (IPSCs) were recorded from the same neuron and evoked by a bipolar electrode (0.2 ms pulses) at the holding potential of − 70 mV and + 10 mV, respectively. EPSCs and IPSCs were quantified by the amplitude of the peak. All EPSCs used for analysis were averaged from 6 consecutive traces with a stimulus interval of 10 s. Data were analyzed using Clampfit 10 software.

For paired-pulse stimulation experiments, the stimulus intensity was set at a level that could evoke a response of 50% saturation for the individual cell measured and delivered with an inter-stimulus interval of 20, 50, 100, and 200 ms. The paired-pulse ratio (PPR) was calculated with the peak current response to the second pulse divided by that of the first response.

### Biocytin staining

Biocytin filling and staining were performed as described previously [24]. To fully infuse the entire cell with the dye, electrophysiological recording lasted for more than 15 min. After electrophysiological recording, brain slices were fixed in neutrally buffered 4% Paraformaldehyde (PFA) solution at 4 °C overnight, washed three times in phosphate buffer saline (PBS) and then, stained with streptavidin-Cy3 (1:100 of 1 mg/ml, S6402, Sigma) for 3 h at room temperature and mounted on glass coverslips for visualization of the biocytin-filled neurons.

### Analysis of dendrites and dendritic spines

Image acquisition was carried out using a Leica SP8 confocal microscope and Leica Application Suite X software (ver.1.4.5, Leica, Germany). Images were acquired at a resolution of 1024 or 2048 pixels in the X–Y dimension. The Z dimensions were variable. Most of the neurons were photographed using a 10 × objective lens for the analysis of dendritic branches. The Z-dimensional increment was 2 µm for Z-stack images. A 100 × objective lens was used for dendritic spine imaging. For the spine Z-stack images, the Z-dimensional increment ranged between 0.1 and 0.3 µm. The stack images were analyzed using Imaris software (version 9.5.1, Bitplane, Switzerland). 3D reconstruction of a single neuron was performed to remove the interference of surrounding neuronal dendrites and the total length of dendrites was automatically calculated. For the Sholl analysis, the spheres were constructed continuously from the center of the cell body with the radius increased by 10 µm. The number of intersections between each circle and dendrites was calculated for comparison.

To compare the difference in spine density between the WT and mutant dogs, spines in secondary dendrites were chosen for the analysis. The spines in the dendrites were detected by Imaris software semi-automatically. The mislabeled spines were deleted manually. Spine dimensions were defined by the length of the spine and the width of the spine neck and the spine head, which allowed to classify the spines into four types: stubby, mushroom, long thin, and filopodia. The stubby type had a length < 1 µm; the mushroom type had a length > 3 µm, and the maximum width of the head/the mean of the neck was > 2; the long thin type had a ratio of the mean width of the head/mean width of the neck ranging from 1 to 2, and the rest of the spines were filopodia. Spine measurements were performed using MATLAB-XTension Spines Classifier in Imaris.

### Golgi staining

Immediately after anesthesia, the PFC tissue was removed, washed with 0.1 M PBS (4 °C), and cut into segments of 1 cm^3^. Following the instructions of a commercial Golgi staining kit (FD NeuroTechnologies, Inc., Columbia, MD, USA), the brain tissue was placed in a mixture of equal amounts of solution A and solution B, then stored in the dark at room temperature for 14 days. Afterward, the tissue was soaked in solution C and stored at room temperature for another 3 days. PFC tissue sections (100 μm) were made under a cryomicroscope (Leica CM1950, Germany) and placed in a mixture of solution D, solution E, and distilled water (1:1:2) for 10 min. The sections were rinsed in distilled water and then dehydrated with various gradients of ethanol (50, 75, and 95%) for 5 min each, then rinsed with xylene and sealed with resin glue. After 24 h in the dark and under ventilated condition, the slices were visualized using a bright-field microscope (Zeiss LSM980 with Airyscan2, Germany). To quantify spine density, images of at least 10–15 neurons in the PFC for each brain were taken. The number and length of dendritic spines along a 100 μm secondary dendrite were recorded using Image J software.

### Transmission electron microscopy

The sample for ultrastructure analysis was prepared as described previously [25]. Briefly, fresh dog PFC slices were prepared as described above. Brain slices were then fixed with 2.5% glutaraldehyde with 0.1 M Phosphate Buffer (PB) buffer (pH 7.4) for at least 30 min at room temperature. Layer 2/3 PFC was dissected out from the brain slices under a stereoscope. Blocks were then post-fixed for 2 h with 1% osmium tetraoxide in PB for 2 h at 4 °C, dehydrated through a graded ethanol series (30, 50, 70, 80, 90, 100%, 100%, 7 min each) into pure acetone (2 × 10 min). Samples were infiltrated in a graded mixture (3:1, 1:1, 1:3) of acetone and SPI-Pon 812 resin (16.2 g SPI-Pon 812 epoxy resin monomer, 10 g dodecenyl succinic anhydride, and 8.9 g nadic methyl anhydride), then changed to pure resin. Finally, tissues were embedded in pure resin with 1.5% N, N-dimethylbenzylamine and polymerized for 12 h at 45 °C, and 48 h at 60 °C. The ultrathin sections (70 nm thickness) were sectioned with a microtome (Leica EM UC6), and collected on single-slot formvar-coated copper grids. The sections were then doubly stained with uranyl acetate and lead citrate. Images were acquired at 30,000 × magnification using a transmission electron microscope (FEI Tecnai Spirit120kV). Analysis of secretory vesicles and postsynaptic density measurements was performed using Image J by an observer who was blinded to the genotype of samples. The active zone was defined by apposition to the PSD, and vesicles in the active zone were defined as those touching the presynaptic plasma membrane or within a distance of 50 nm to the presynaptic membrane juxtaposing the PSD [26, 27]. Since single sections were used for the ultrastructural analysis, potential errors cannot be excluded.

### Statistical analysis

All collected data except those cells with poor recording quality were used for statistics. Statistical analyses were performed using GraphPad Prism 8.2.1 (GraphPad Software, Inc.). We first analyzed the normality distribution of all datasets by the Shapiro–Wilk test, group differences of normality distribution data were assessed through the two-tailed unpaired t test, and data with non-normality distribution were analyzed by Mann–Whitney test or Kolmogorov–Smirnov test. Data with two independent variables were analyzed using two-way repeated measures ANOVA followed by Bonferroni’s post hoc test. Data are presented as the mean ± SEM for bar graphs or median and quartiles for violin plot. Statistical significance was set at *p* < 0.05.

## Results

### PFC pyramidal neuron hyperexcitability in acute brain slices from *Shank3* mutants

**Fig. 1:**
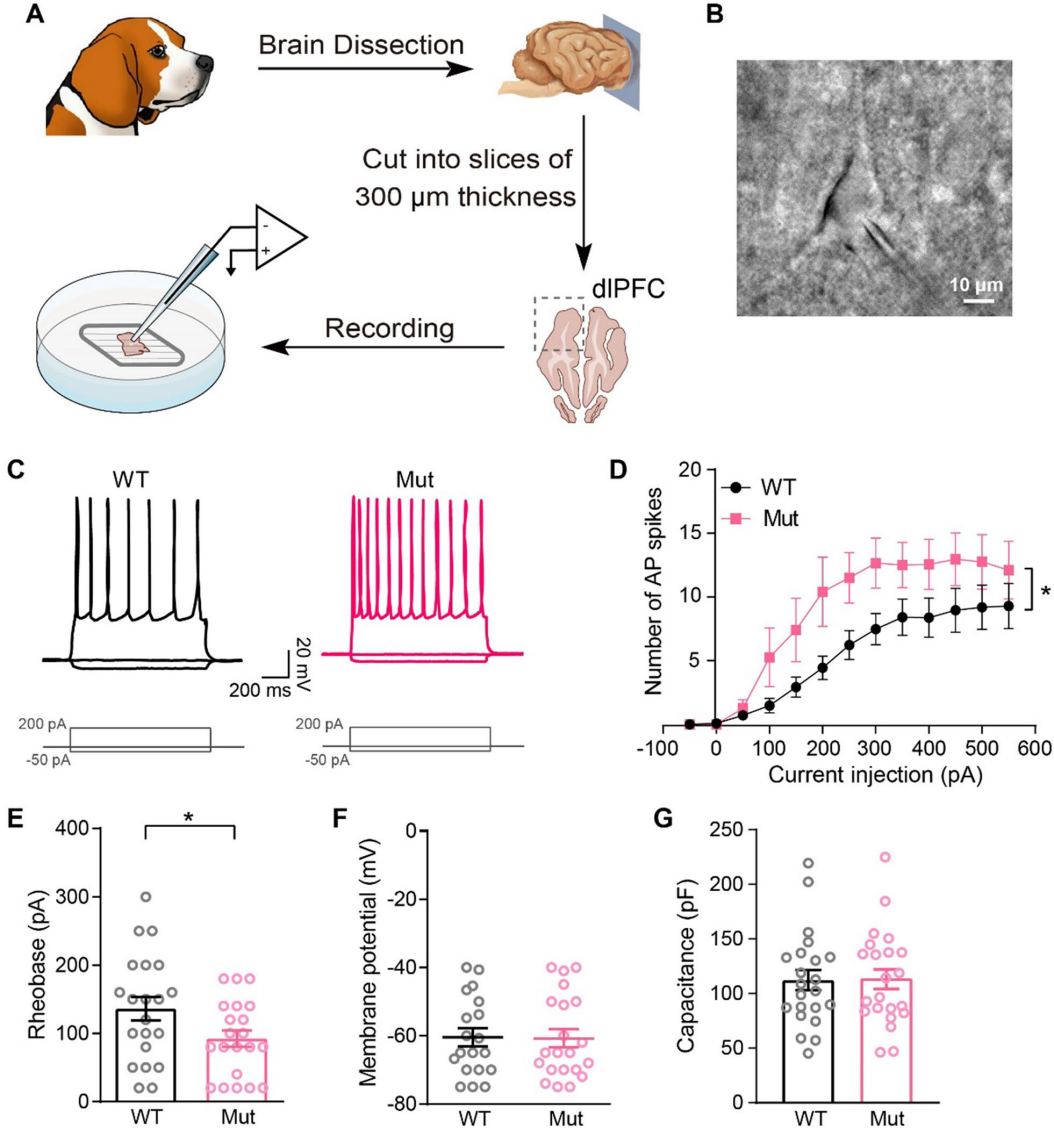
Hyperexcitability of pyramidal neurons in the PFC slices of* Shank3* heterozygous mutant dogs. **A** Schematic illustrating the preparation of neocortex slices and electrophysiological recording. **B** A representative image of a recorded layer 2/3 pyramidal neuron in fresh PFC brain slices. Scale bar, 10 μm. **C** Representative spiking pattern of pyramidal neurons in response to − 50 pA, 0 pA, and + 200 pA current injections. **D–G** Compared with WT controls, PFC pyramidal neurons from *Shank3* mutant dogs displayed **D** a left-shifted current-response curve (WT: *n* = 27 neurons, *Shank3* mutant: *n* = 20 neurons; *p* = 0.0325), **E** decreased rheobase (WT: *n* = 21 neurons, *Shank3* mutant: *n* = 21 neurons; *p* = 0.0437), and no change in **F** resting membrane potential (WT: *n* = 19 neurons, *Shank3* mutant: *n* = 21 neurons; *p* = 0.9359) and **G** capacitance (WT: *n* = 22 neurons, *Shank3* mutant: *n* = 22 neurons; *p* = 0.9371). Data in **E**–**G** were analyzed with a two-tailed, unpaired t test. Data in **D** were analyzed with two-way repeated measure ANOVA. All data were collected from 3 pairs of animals, and presented as mean ± SEM; *p < 0.05, **p < 0.01 and ***p < 0.001

To define the electrophysiological properties of neurons in our autism-associated dog model, we prepared acute brain slices from *Shank3* heterozygous mutants (Mut) and wild-type (WT) dogs, and maintained slices under consistent perfusion in aCSF. A fresh coronal brain slice of dorsal lateral PFC (one of the most studied PFC subregions involving cognitive processes) was transferred to the recording chamber for electrophysiological recordings (Fig. 1A). In slices, pyramidal neurons in layer 2/3 of the PFC were recorded by whole-cell patch clamp. Pyramidal neurons were morphologically recognized under light microscopy and confirmed by biocytin staining injected from a recording pipette (Fig. 1B). To characterize the electrophysiological properties of pyramidal cells, we depolarized neurons with a series of gradient-increasing current injections. Pyramidal neurons showed no spontaneous firing at baseline but initiated action potentials (APs) with a current injection of ≥ 50 pA and reached a maximum firing frequency of approximately 10 Hz (Fig. 1C, D), similar to electrophysiological properties of neocortical pyramidal neurons in humans [28, 29], rodents [30], and nonhuman primates [31].

We then examined the intrinsic excitability of pyramidal cells and found that layer 2/3 pyramidal neurons in *Shank3* mutant dogs were more hyperactive than those in WT dogs, with a left-shifted current-response curve (Fig. 1C, D). Consistently, rheobase currents were decreased (WT: 136.2 ± 17.13 pA, Mut: 92.38 ± 12.07 pA; *p* = 0.0437) (Fig. 1E), while the resting membrane potential and membrane capacitance remained normal in *Shank3* mutants compared to WT controls (Fig. 1F, G), suggesting that intrinsic excitability in pyramidal neurons was increased in *Shank3* mutant dogs. However, AP properties such as AP threshold, AP amplitude, AP rise time, and after hyperpolarization amplitude appeared normal but showed slightly prolonged decay times and half-widths in *Shank3* mutants (Additional file 1: Fig. S1 A–H). These results showed that intrinsic excitability in PFC pyramidal cells was increased in *Shank3* mutant dogs.

### Impaired synaptic transmission of layer 2/3 pyramidal neurons in *Shank3* mutant dogs

**Fig. 2:**
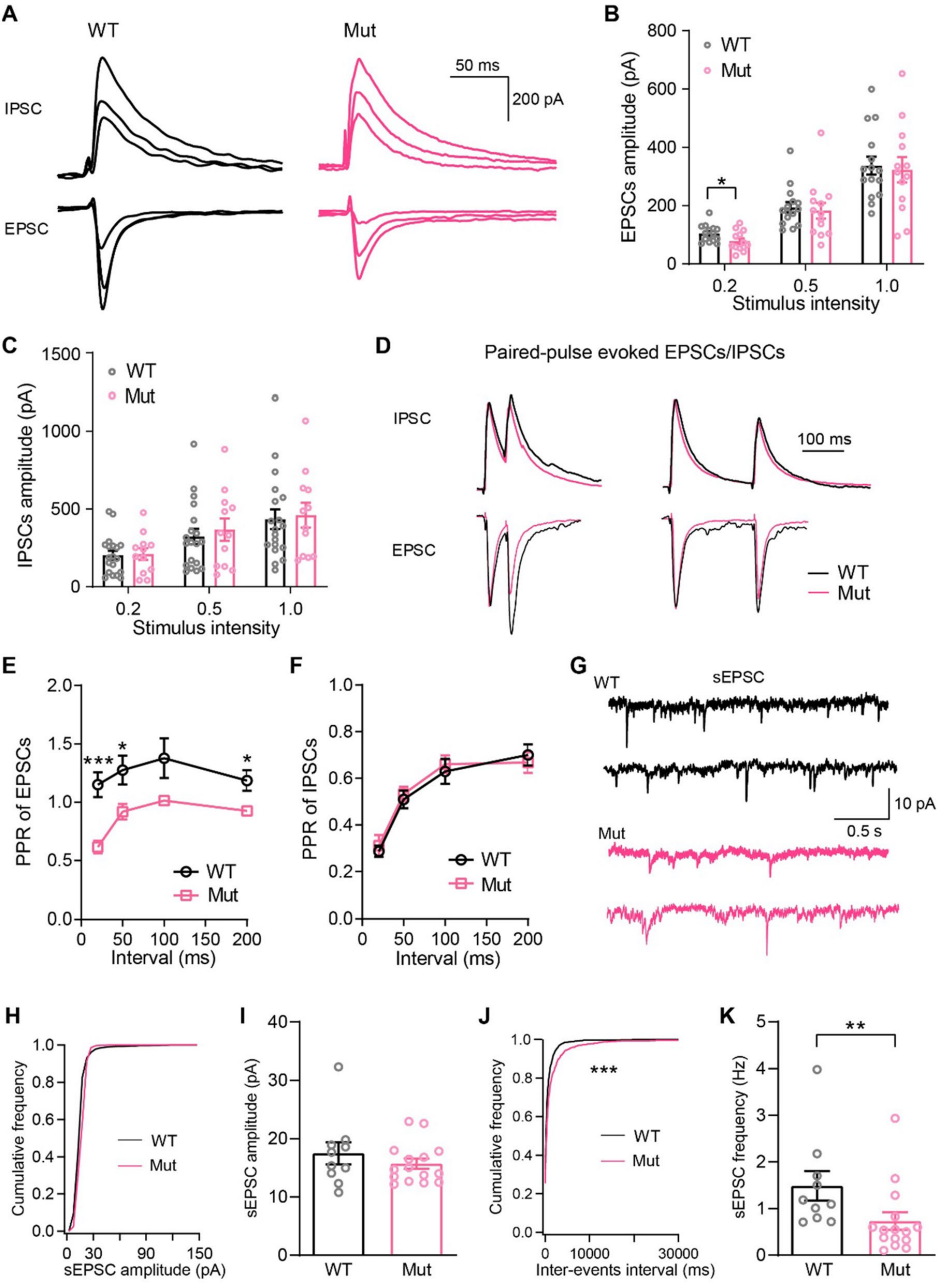
Impaired glutamatergic synaptic transmission of layer 2/3 pyramidal neurons in *Shank3* mutant dogs. **A** The representative EPSCs and IPSCs of PFC pyramidal neurons from WT and *Shank3* mutant dogs. EPSCs and IPSCs were evoked by 20%, 50%, and 100% saturation stimulus. **B** Comparison of EPSCs amplitude between WT and *Shank3* mutant dogs (WT: *n* = 8 neurons, *Shank3* mutant: *n* = 10 neurons; *p* = 0.0351, 0.7186, and 0.7889 for stimulus intensities at 20%, 50%, and 100% saturation levels, respectively). **C** Comparison of IPSCs amplitude between WT and *Shank3* mutant dogs (WT: *n* = 8 neurons, *Shank3* mutant: *n* = 11 neurons; *p* = 0.8698, 0.6016, and 0.7939 for stimulus intensities at 20%, 50%, and 100% saturation levels, respectively). **D** The normalized EPSCs and IPSCs in response to paired pulses with intervals of 50 ms and 200 ms. **E** Paired-pulse ratio of EPSCs of WT and *Shank3* mutant dogs (WT: *n* = 14 neurons, *Shank3* mutant: *n* = 12 neurons; *p* = 0.0003, 0.0236, 0.0648, and 0.0159 for paired-pulses intervals at 20 ms, 50 ms,100 ms, and 200 ms, respectively). **F** Paired-pulse ratio of IPSCs of WT and *Shank3* mutant dogs (WT: *n* = 13 neurons, *Shank3* mutant: *n* = 13 neurons; *p* = 0.6568, 0.6435, 0.6527, and 0.6097 for paired-pulses intervals at 20 ms, 50 ms,100 ms, and 200 ms, respectively). **G** Representative spontaneous EPSCs (sEPSCs) of PFC pyramidal neurons from WT and *Shank3* mutant dog. **H, J** Cumulative distributions of amplitude and inter-events interval of sEPSCs (WT: *n* = 10 neurons, *Shank3* mutant: *n* = 15 neurons; *p* < 0.0001). **I, K** The histograms for the amplitude (I) and frequency (K) of sEPSCs. Data in **B, C, E, F, I** and** K** were analyzed with a two-tailed, unpaired t test. Data in **H** and** J** were analyzed with Kolmogorov–Smirnov test. All data were collected from 3 pairs of animals, and presented as mean ± SEM; *p < 0.05, **p < 0.01 and ***p < 0.001

Previous studies have reported impaired synaptic functions which were hypothesized as mechanism underlying behavioral abnormalities in *Shank3* mutant rodents [2, 7, 30, 32–35]. To determine if synaptic function was impaired in *Shank3* mutant dogs, we examined evoked excitatory postsynaptic current (EPSC) amplitudes in response to different stimulus intensities (20%, 50%, and 100% saturation). We found that EPSCs in layer 2/3 pyramidal neurons in *Shank3* mutants showed reduced trends when compared to WT controls. In particular, EPSCs in response to lower stimulus intensity (20% saturation) were significantly decreased in *Shank3* mutants when compared to WT controls (WT: 104.6 ± 7.47 pA, Mut: 78.27 ± 9.34 pA;* p* = 0.0351), suggesting impaired glutamatergic synaptic transmission in pyramidal neurons (Fig. 2A, B). However, inhibitory postsynaptic currents (IPSCs) in *Shank3* mutant dogs in response to various stimulus intensities were comparable to WT dogs (Fig. 2A, C).

We then examined presynaptic release probability by calculating paired-pulse ratios (PPRs) of EPSCs and IPSCs. The PPR, which is the ratio of the amplitude of the second response to that of the first, has been successfully used to calculate vesicular release probability at presynapses [36]. While the PPR of IPSCs did not change, the PPR of EPSCs in *Shank3* mutant dogs was lower than WT dogs, indicating an enhanced presynaptic glutamate release probability (Fig. 2D–F). This likely occurred as a side-effect of presynapses compensating for long-term postsynaptic deficiency. Furthermore, the amplitude of spontaneous EPSCs (sEPSCs) was not affected but the sEPSC frequency was decreased due to *Shank3* loss (Fig. 2G–K). Taken together, these results suggested that glutamatergic synaptic transmission onto layer 2/3 pyramidal neurons in the PFC was impaired in *Shank3* mutant dogs.

### Reduced dendrite complexity and spine density in *Shank3* mutant dogs

**Fig. 3:**
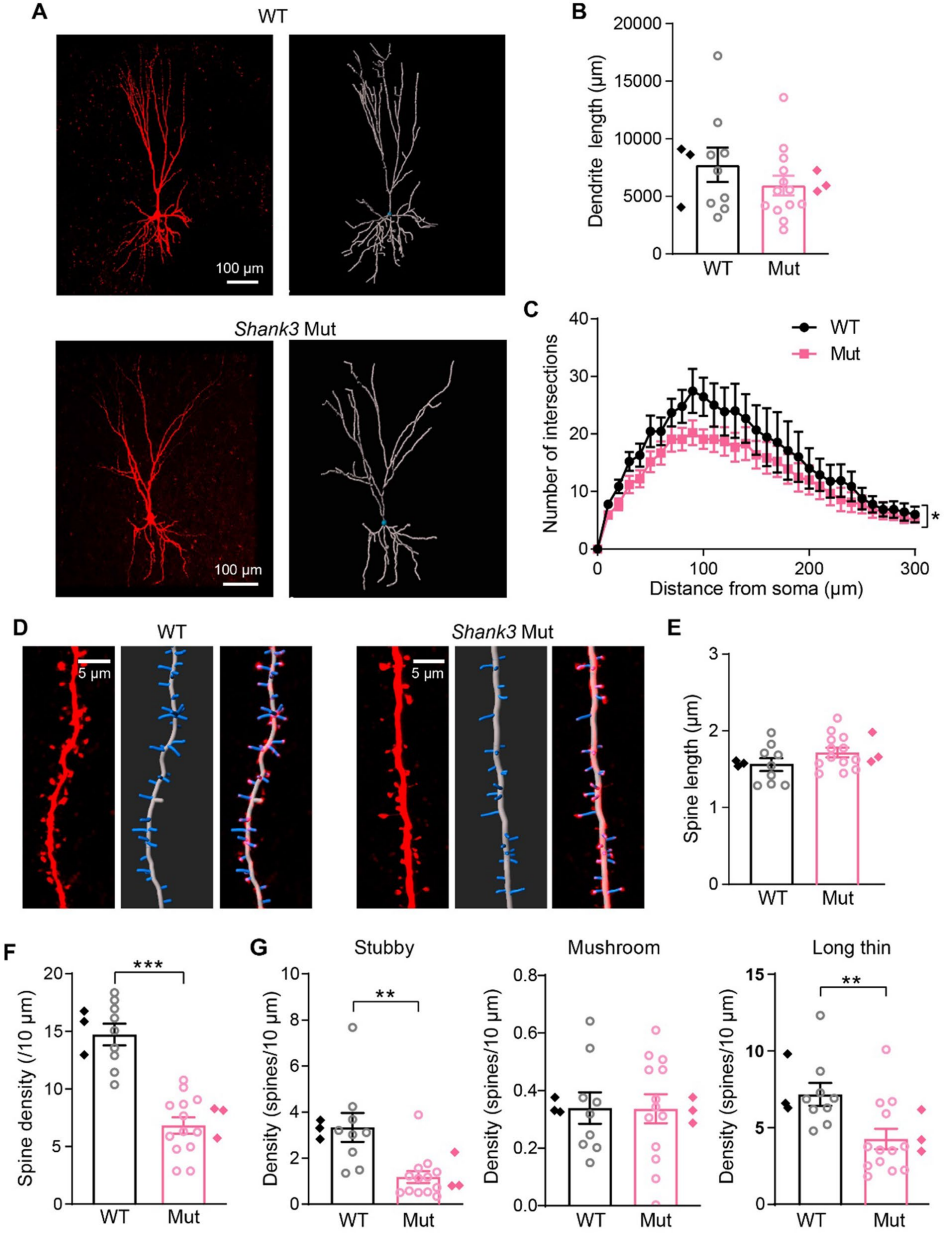
Decreased dendrite complexity and spine density in the PFC of *Shank3* mutant dogs. **A** Representative images (left panel) and 3D reconstruction (right panel) of biocytin-filled pyramidal neurons in the layer 2/3 PFC from *Shank3* mutant and WT dogs. The neurons were filled with biocytin via patch pipette and stained with streptavidin-Cy3. Scale bars, 100 μm. **B** Analysis of the total dendrites length of pyramidal neurons from *Shank3* mutant and WT dogs (WT: *n* = 9 neurons, *Shank3* mutant: *n* = 13 neurons; *p* = 0.2767). **C** Sholl analysis of dendritic branching complexity in the PFC pyramidal neurons from WT and mutant dogs (WT: *n* = 9 neurons, *Shank3* mutant: *n* = 13 neurons; *p* = 0.0494). **D** The representative images showing the dendritic spines of layer 2/3 pyramidal neurons from WT and *Shank3* mutant dogs: the confocal slice (left), 3D reconstruction of spines (middle), and the merged image (right). Scale bar, 5 μm. **E** Averaged spine length of pyramidal neurons obtained from WT and *Shank3* mutant dogs (WT: *n* = 9 neurons, *Shank3* mutant: *n* = 13 neurons; *p* = 0.1620). **F** The spine density on the dendrites of pyramidal neurons obtained from WT and *Shank3* mutant dogs (WT: *n* = 9 neurons, *Shank3* mutant: *n* = 13 neurons; *p* < 0.0001). **G** Comparisons of densities for stubby (*p* = 0.0011), mushroom (*p* = 0.9729), and long thin spines (*p* = 0.0056) between WT and *Shank3* mutant dogs. Data in **B**, **E**, and** F** were analyzed with a two-tailed, unpaired t test. Data in **C** and **G** were analyzed with Mann–Whitney test. All data were collected from 3 pairs of animals. Data points represent neurons (circle) and dogs (rhombus) for each group, and presented as mean ± SEM; *p < 0.05, **p < 0.01 and ***p < 0.001

Neocortical pyramidal neurons are morphologically characterized by distinct basal and apical dendrites that receive distinctly organized synaptic inputs from different afferents [37]. The aforementioned synaptic deficits in this study may have been caused by changes in dendrite elaboration or spine morphology and density brought on by *Shank3* mutations. To determine if neurons showed morphological changes in *Shank3* mutant dogs, pyramidal neurons were filled with biocytin during electrophysiological recordings and counterstained with streptavidin-Cy3. High-resolution confocal z-stack scans were acquired (Fig. 3A), stitched with ImageJ, and digitally reconstructed using Imaris software for analyzing the dendritic spine density and morphology (Table 1).

**Table 1:** Numbers of animals, neurons, dendrites, spines, and synapses examined in the morphological and EM analysis

	WT	*Shank3* Het
Animals	3 males	2 males, 1 female
Neurons	9 (4, 3, 2)	13 (7, 3, 3)
Dendrites	55 (32, 13, 10)	69 (34, 18, 17)
Total spines	14,173 (5497, 3870, 4806)	8251 (3863, 2349, 2039)
Thin	8869 (3373, 2334, 3162)	5412 (2500, 1462, 1450)
Stubby	4133 (1600, 1179, 1354)	1740 (875, 620, 245)
Mushroom	662 (281, 257, 124)	717 (341, 176, 200)
Filopodia	509 (243, 100, 166)	382 (147, 91, 144)
Synapses	228 (94, 82, 52)	213 (57, 87, 69)

Data show total counts for each category, the numbers in the parentheses correspond to three different animals

Labeled neurons presented with a typical pyramidal neuronal shape with distinct apical dendrites, extensive basal dendritic trees, and spines on dendrites. Total dendrite length was quantified for neurons, with no significant differences in dendritic length between *Shank3* mutant and WT dogs (WT: 7,730 ± 1489 µm, Mut: 5,936 ± 856.5 μm,* p* = 0.2767; Fig. 3B). However, Sholl analysis at 10 µm incremental distances from the cell soma showed that the number of dendrite intersections in pyramidal neurons from *Shank3* mutants was decreased when compared to WT dogs (Fig. 3C), indicating reduced dendritic complexity and consequently reduced synaptic connections with presynaptic terminals. Although averaged spine length was not significantly altered (WT: 1.57 ± 0.08 μm, Mut: 1.71 ± 0.06 μm,* p* = 0.1620; Fig. 3D, E), total dendritic spine density in *Shank3* mutant dogs was strikingly lower than that of WT dogs (WT: 14.71 ± 0.94/10 μm, Mut: 6.82 ± 0.71/10 μm,* p* < 0.0001; Fig. 3D, F). Similar results were also observed in PFC pyramidal neurons labeled by Golgi staining (Additional file 1: Fig. S2 A, B). These results together showed that PFC pyramidal neurons exhibited neuronal structural deficits in *Shank3* mutant dogs, consistent with our findings of impaired synaptic transmission onto pyramidal neurons (Fig. 2).

Dendritic spines are classified as thin, stubby, mushroom, and filopodia [38, 39] structures based on their length and the relative size of their head and neck. Mushroom spine density (WT: 0.34 ± 0.05/10 μm, Mut: 0.33 ± 0.05/10 μm;* p* = 0.9729) was not significantly altered in *Shank3* mutant dogs when compared to WT dogs, but stubby spine (WT: 3.30 ± 0.63/10 μm, Mut: 1.14 ± 0.26/10 μm;* p* = 0.0011) and long thin spine (WT: 7.17 ± 0.75/10 μm, Mut: 4.27 ± 0.67/10 μm;* p* = 0.0056) densities were remarkably reduced (Fig. 3G). As few filopodia spines were available, a statistical analysis was not conducted. Notably, we used 3–4-month-old juvenile dogs for experiments because autism has a high incidence in childhood. At this age, we observed more stubby and long thin spines when compared with mushroom spines in dog PFC, consistent with the notion that stubby spines are predominant during early postnatal development in mice [40].

### Excitatory synapse PSD is smaller in the PFC of *Shank3* mutant dogs

**Fig. 4:**
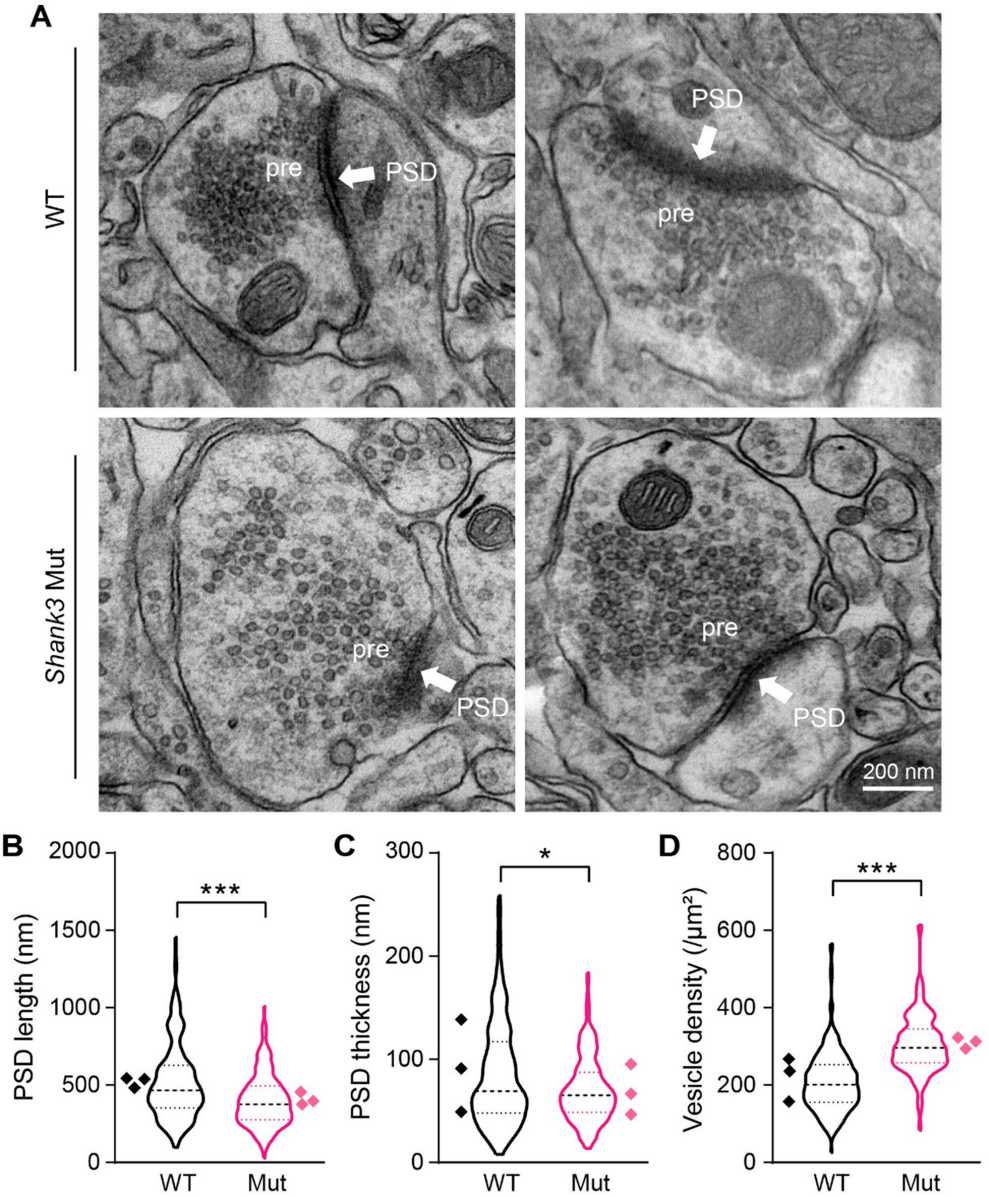
Smaller PSD of the excitatory synapse in *Shank3* mutant dogs. **A** Electron microscope images of excitatory synapses in layer 2/3 of PFC. White arrows indicate postsynaptic densities (PSD). Pre, presynapse; scale bar 200 nm. **B** PSDs are shorter in *Shank3* mutants compared with WT controls (*p* < 0.0001). **C** PSDs are thinner in *Shank3* mutants compared with WT controls (*p* = 0.0227). **D** Increased density of presynaptic vesicles at the excitatory synapses in mutant dog (*p* < 0.0001). Data in **B**–**D** were analyzed with Mann–Whitney test. *n* = 228 synapses from WT dogs, and *n* = 213 synapses from *Shank3* mutants, data are presented as median and quartiles. The data points for each dog are presented as rhombus, *p < 0.05, **p < 0.01, and ***p < 0.001

To further examine neuron morphological changes in *Shank3* mutant dogs, we examined excitatory synapse ultrastructure by electron microscopy (Table 1). Excitatory synapses were well-recognized by asymmetric pre- and postsynaptic structures. Each excitatory synapse was characterized by a cluster of round presynaptic vesicles and a robust PSD, which is an electron-dense thickening underneath postsynaptic membranes [41]. We observed that PSDs were shorter (WT: 517.5 ± 16.2 nm, Mut: 399.7 ± 11.6 nm;* p* < 0.0001) and thinner (WT: 85.0 ± 3.3 nm, Mut: 70.5 ± 2.2 nm;* p* = 0.0003) in *Shank3* mutant dogs when compared to WT dog (Fig. 4A–C). However, *Shank3* mutant dogs showed a higher vesicle density in the active zone of excitatory synapses (WT: 209.0 ± 5.2/µm^2^, Mut: 304.9 ± 5.3/μm^2^, *p* < 0.0001; Fig. 4D), in agreement with increased vesicle release probability (Fig. 2D, E) and were likely generated as a result of compensatory mechanisms for long-term synaptic deficits.

## Discussion

*Shank3* mutant dogs exhibit autism-like social deficits, including social withdrawal, elevated anxiety, and greater sensitivity to deviant tones [13, 14]. In the present study, we found that the synaptic transmission of layer 2/3 pyramidal neurons in the PFC was decreased, and accompanied by reduced dendritic spine density in *Shank3* mutant dogs when compared to WT dogs. Additionally, pyramidal neuron excitability was enhanced in *Shank3* mutant dogs. These results suggested that PFC pyramidal neuron morphologies and functions were altered by *Shank3* haploinsufficiency, which may be an underlying mechanism of abnormal behaviors observed in *Shank3* mutant dogs [13, 14].

Electrophysiological recording in acute brain slices from mammalian brains is an important technique for studying neuronal activity and synaptic connections as an alternative to in vivo recordings [42, 43]. However, due to experimental animal limitations arising from ethical considerations, electrophysiological recordings, especially patch clamp, are rarely applied to large animals [28, 44, 45]. Here, for the first time, we prepared acute PFC brain slices from dogs following rodent protocols and performed whole-cell recordings on single pyramidal neurons. The majority of recorded neurons were in good condition as measured by regular spiking patterns, resting membrane potentials, and synaptic responses. The electrophysiological properties of canine PFC layer 2/3 pyramidal neurons were similar to rodents and primates [8, 28, 30, 31]. This work demonstrated the feasibility of electrophysiological recordings in brain slices to reveal physiological and pathological neuronal properties in canines.

We observed that PFC pyramidal neurons from *Shank3* mutant dogs exhibited higher excitability than WT animals (Fig. 1C, D), along with reduced rheobase currents (Fig. 1E). Similar results were previously reported in striatal medium spiny neurons from *Shank3* null mice carrying a deletion of protein-coding exons 4–22 [46], layer 2/3 pyramidal neurons in medial PFC from a *Shank3* mouse line with deleted exons 14–16 [30], and human neurons harboring a *SHANK3* (exon 13) deletion [47]. These findings, together with increased neuronal excitability in PFC layer 2/3 neurons from *Shank3* mutant dogs observed in our study, suggested that increased neuronal excitability was a conserved phenotype caused by *Shank3* deletions. However, how *Shank3* is implicated in neuronal excitability regulation remains an enigma. Yi et al*.* [47] provided evidence that *SHANK3* haploinsufficiency caused dysfunction of the hyperpolarization-activated cyclic nucleotide-gated channel (HCN) in cell membranes as the SHANK3 protein directly bound to HCN proteins, which in turn caused hyperexcitability in human neurons. Further studies are required to clarify the relationships between decreased synaptic transmission and increased neuronal excitability caused by *Shank3* loss. Additionally, it will be important to determine if the nonsynaptic effects of increased neuronal excitability contribute to autistic behaviors.

Excitatory synaptic transmission onto PFC layer 2/3 pyramidal neurons and spine density were impaired in *Shank3* mutant dogs (Figs. 2 and 3), consistent with the presence of SHANK3 in excitatory postsynaptic specializations where the protein acts as a scaffold to coordinate receptor signaling [48, 49]. Similar results were previously reported in different brain regions (PFC, hippocampus, striatum, or cerebellum) in multiple *Shank3* deficient cell or animal models of different species, including humans, macaques, mice, and rats [3, 8, 50–53]. Although synaptic impairments are hallmarks of *Shank3* deficiency, not all *Shank3* deficient animal models showed this phenotype. For instance, when compared with controls, no changes in dendrite complexity and spine density were observed in medial PFC pyramidal neurons in homozygous and heterozygous *Shank3* deficient rats [54], nor in the hippocampus of *Shank3* mutant mice carrying an exon 21 deletion [55]. Whether synaptic connections are altered in *Shank3* deficient animals may depend on animal age, the brain region, cell type, and the degree and dose of the *Shank3* deletion.

*Shank3* mutant dogs in our study represented hypomorphs where the proline-rich domain encoded by exon 21 of *Shank3* was disturbed. Exon 21 is the largest coding exon of *SHANK3*. Rare pathogenic single nucleotide variants within exon 21 have been reported in individuals with ASD [56, 57]. Both mouse and macaque models, with various exon 21 mutations, showed impaired social interactions [55, 58]. In addition to homozygous *Shank3* deficient mice with exon 21 deletion, heterozygous *Shank3*-deficient mice (*Shank3*^+/ΔC^) also exhibited reduced social behaviors and impaired synaptic transmission, albeit less severe than homozygous mutant phenotypes [35, 59, 60]. Our work has confirmed that *Shank3* haploinsufficiency induced morphological and functional abnormalities in *Shank3* mutant dogs. As ASD patients, including patients with Phelan-McDermid syndrome, typically carries heterozygous *SHANK3* mutations [61, 62], *Shank3* heterozygous mutant animals may better mimic the pathophysiology and behavioral abnormalities of ASD patients.

Dogs at the age of 3–4 months were analyzed in the present study. There are two reasons for this: the brain slices from the younger animals are easier for electrophysiological recordings than from adults; the other is, 3–4 months beagle dogs are in their childhood, and during this time, ASD occurs in humans. According to a recent study [63], the 3–4 months old dogs correspond to human childhood. Therefore, we believe that the dog age of 3–4 months is an appropriate time for the present study because it captures the high occurrence of ASD in children and is also when synaptic development tends to stabilize. We note, however, the brains of individuals with ASD at different age groups undergo different changes as previously reported [19, 64]. For animal welfare and practical considerations, we can only choose animals from a certain age group or developmental stage for comparative research at the moment. As a result, we are unable to determine whether our findings apply to the autistic dog models at other ages.

## Limitations

We identified altered PFC pyramidal neuron morphologies and functions in *Shank3* mutant dogs, but it remains unclear how PFC neuropathology is linked to behavioral alterations. Future work should address this question by manipulating neuronal activity or restoring synaptic transmission in the PFC of *Shank3* mutant dogs. Because *Shank3*’s function in synaptic transmission regulation varies depending on the brain region and neural development stage, spatiotemporal neuropathology features and social behaviors need to be further defined.

## Conclusions

Collectively, pyramidal neuron hyperexcitability, reduced dendrite elaboration and spine densities, and impaired synaptic transmission showed that *Shank3* haploinsufficiency induced morphological and functional abnormalities, providing neurobiological insights underlying social behavior abnormalities. Our work also demonstrated the feasibility of using canine brain slices as a model system to study neuronal circuitry and disease, thereby shedding new light on neuronal mechanisms underlying ASD-associated behaviors.

### Supplementary Information

**Additional file 1:** Supplementary table and figures. **Table S1.** List of juvenile Shank3 mutant dogs used in the present study. **Figure S1.** Action potential properties of PFC pyramidal neurons in Shank3 mutants and WT dogs. **Figure S2.** Decreased spine density in the PFC of Shank3 mutant dogs by Golgi staining.

## Data Availability

The datasets used and/or analyzed in the current study are available from the corresponding author upon request.

## Abbreviations

ASD: Autism spectrum disorder
PFC: Prefrontal cortex
PSD: Postsynaptic density
AP: Action potential
EPSCs: Evoked excitatory postsynaptic currents
IPSCs: Inhibitory postsynaptic currents
PPR: Paired-pulse ratio
PFA: Paraformaldehyde
PBS: Phosphate buffer saline
sEPSCs: Spontaneous EPSCs
HCN: Hyperpolarization-activated cyclic nucleotide-gated channel
Mut: Mutant
WT: Wide type

## References

[1] Sheng M, Kim E (2000). The shank family of scaffold proteins. J Cell Sci.

[2] Delling JP, Boeckers TM (2021). Comparison of Shank3 deficiency in animal models: phenotypes, treatment strategies, and translational implications. J Neurodev Disord.

[3] Peca J, Feliciano C, Ting JT, Wang W, Wells MF, Venkatraman TN, Lascola CD, Fu Z, Feng G (2011). Shank3 mutant mice display autistic-like behaviours and striatal dysfunction. Nature.

[4] Wang W, Li C, Chen Q, van der Goes MS, Hawrot J, Yao AY, Gao X, Lu C, Zang Y, Zhang Q (2017). Striatopallidal dysfunction underlies repetitive behavior in Shank3-deficient model of autism. J Clin Invest.

[5] Harony-Nicolas H, Kay M, du Hoffmann J, Klein ME, Bozdagi-Gunal O, Riad M, Daskalakis NP, Sonar S, Castillo PE, Hof PR (2017). Oxytocin improves behavioral and electrophysiological deficits in a novel Shank3-deficient rat. Elife.

[6] Barak B, Feng G (2016). Neurobiology of social behavior abnormalities in autism and Williams syndrome. Nat Neurosci.

[7] Mei Y, Monteiro P, Zhou Y, Kim JA, Gao X, Fu Z, Feng G (2016). Adult restoration of Shank3 expression rescues selective autistic-like phenotypes. Nature.

[8] Guo BL, Chen J, Chen Q, Ren KK, Feng DY, Mao HH, Yao H, Yang J, Liu HY, Liu YY (2019). Anterior cingulate cortex dysfunction underlies social deficits in Shank3 mutant mice. Nat Neurosci.

[9] Bunford N, Andics A, Kis A, Miklosi A, Gacsi M (2017). Canis familiaris as a model for non-invasive comparative neuroscience. Trends Neurosci.

[10] MacLean EL, Herrmann E, Suchindran S, Hare B (2017). Individual differences in cooperative communicative skills are more similar between dogs and humans than chimpanzees. Anim Behav.

[11] Topal J, Roman V, Turcsan B (2019). The dog (canis familiaris) as a translational model of autism: it is high time we move from promise to reality. Wiley Interdiscip Rev Cogn Sci.

[12] Spocter MA, Uddin A, Ng JC, Wong E, Wang VX, Tang C, Wicinski B, Haas J, Bitterman K, Raghanti MA (2018). Scaling of the corpus callosum in wild and domestic canids: insights into the domesticated brain. J Comp Neurol.

[13] Tian R, Li Y, Zhao H, Lyu W, Zhao J, Wang X, Lu H, Xu H, Ren W, Tan QQ (2023). Modeling Shank3-associated autism spectrum disorder in beagle dogs via crispr/cas9 gene editing. Mol Psychiatry.

[14] Wu L, Mei S, Yu S, Han S, Zhang YQ (2023). Shank3 mutations enhance early neural responses to deviant tones in dogs. Cereb Cortex.

[15] Fuster JM (2001). The prefrontal cortex—an update: time is of the essence. Neuron.

[16] Yan Z, Rein B (2022). Mechanisms of synaptic transmission dysregulation in the prefrontal cortex: pathophysiological implications. Mol Psychiatry.

[17] Yu B, Yuan B, Dai JK, Cheng TL, Xia SN, He LJ, Yuan YT, Zhang YF, Xu HT, Xu FQ (2020). Reversal of social recognition deficit in adult mice with mecp2 duplication via normalization of mecp2 in the medial prefrontal cortex. Neurosci Bull.

[18] Jacot-Descombes S, Uppal N, Wicinski B, Santos M, Schmeidler J, Giannakopoulos P, Heinsein H, Schmitz C, Hof PR (2012). Decreased pyramidal neuron size in brodmann areas 44 and 45 in patients with autism. Acta Neuropathol.

[19] Courchesne E, Mouton PR, Calhoun ME, Semendeferi K, Ahrens-Barbeau C, Hallet MJ, Barnes CC, Pierce K (2011). Neuron number and size in prefrontal cortex of children with autism. JAMA.

[20] Qin L, Ma K, Yan Z (2019). Chemogenetic activation of prefrontal cortex in Shank3-deficient mice ameliorates social deficits, nmdar hypofunction, and sgk2 downregulation. iScience.

[21] Reyes AD (2019). Neuronal signals thoroughly recorded. Nature.

[22] Edwards FA, Konnerth A, Sakmann B, Takahashi T (1989). A thin slice preparation for patch clamp recordings from neurones of the mammalian central nervous system. Pflugers Arch.

[23] Palazzi X (2011). The beagle brain in stereotaxic coordinates.

[24] Swietek B, Gupta A, Proddutur A, Santhakumar V (2016). Immunostaining of biocytin-filled and processed sections for neurochemical markers. J Vis Exp.

[25] Zhu F, Liu L, Li J, Liu B, Wang Q, Jiao R, Xu Y, Wang L, Sun S, Sun X (2022). Cocaine increases quantal norepinephrine secretion through net-dependent pkc activation in locus coeruleus neurons. Cell Rep.

[26] Tan C, Wang SSH, de Nola G, Kaeser PS (2022). Rebuilding essential active zone functions within a synapse. Neuron.

[27] Tyler WJ, Pozzo-Miller LD (2001). Bdnf enhances quantal neurotransmitter release and increases the number of docked vesicles at the active zones of hippocampal excitatory synapses. J Neurosci.

[28] Berg J, Sorensen SA, Ting JT, Miller JA, Chartrand T, Buchin A, Bakken TE, Budzillo A, Dee N, Ding SL (2021). Human neocortical expansion involves glutamatergic neuron diversification. Nature.

[29] Schwarz N, Uysal B, Welzer M, Bahr JC, Layer N, Loffler H, Stanaitis K, Harshad PA, Weber YG, Hedrich UBS (2019). Long-term adult human brain slice cultures as a model system to study human cns circuitry and disease. Elife.

[30] Yoo T, Cho H, Park H, Lee J, Kim E (2019). Shank3 exons 14–16 deletion in glutamatergic neurons leads to social and repetitive behavioral deficits associated with increased cortical layer 2/3 neuronal excitability. Front Cell Neurosci.

[31] Piette C, Vandecasteele M, Bosch-Bouju C, Goubard V, Paille V, Cui Y, Mendes A, Perez S, Valtcheva S, Xu H (2021). Intracellular properties of deep-layer pyramidal neurons in frontal eye field of macaque monkeys. Front Synaptic Neurosci.

[32] Yang M, Bozdagi O, Scattoni ML, Wohr M, Roullet FI, Katz AM, Abrams DN, Kalikhman D, Simon H, Woldeyohannes L (2012). Reduced excitatory neurotransmission and mild autism-relevant phenotypes in adolescent Shank3 null mutant mice. J Neurosci.

[33] Speed HE, Kouser M, Xuan Z, Reimers JM, Ochoa CF, Gupta N, Liu SN, Powell CM (2015). Autism-associated insertion mutation (InsG) of exon 21 causes impaired synaptic transmission and behavioral deficits. J Neurosci.

[34] Zhou Y, Kaiser T, Monteiro P, Zhang XY, Van der Goes MS, Wang DQ, Barak B, Zeng ML, Li CC, Lu CY (2016). Mice with mutations associated with ASD and schizophrenia display both shared and distinct defects. Neuron.

[35] Qin L, Ma K, Wang ZJ, Hu Z, Matas E, Wei J, Yan Z (2018). Social deficits in Shank3-deficient mouse models of autism are rescued by histone deacetylase (HDAC) inhibition. Nat Neurosci.

[36] Branco T, Staras K (2009). Perspectives the probability of neurotransmitter release: variability and feedback control at single synapses. Nat Rev Neurosci.

[37] van Aerde KI, Feldmeyer D (2015). Morphological and physiological characterization of pyramidal neuron subtypes in rat medial prefrontal cortex. Cereb Cortex.

[38] Son J, Song S, Lee S, Chang S, Kim M (2011). Morphological change tracking of dendritic spines based on structural features. J Microsc.

[39] Rodriguez A, Ehlenberger DB, Dickstein DL, Hof PR, Wearne SL (2008). Automated three-dimensional detection and shape classification of dendritic spines from fluorescence microscopy images. PloS One.

[40] Yuste R (2010). Dendritic spines.

[41] Bloss EB, Puri R, Yuk F, Punsoni M, Hara Y, Janssen WG, McEwen BS, Morrison JH (2013). Morphological and molecular changes in aging rat prelimbic prefrontal cortical synapses. Neurobiol Aging.

[42] Manz KM, Siemann JK, McMahon DG, Grueter BA (2021). Patch-clamp and multi-electrode array electrophysiological analysis in acute mouse brain slices. STAR Protoc.

[43] Whitebirch AC, LaFrancois JJ, Jain S, Leary P, Santoro B, Siegelbaum SA, Scharfman HE (2022). Enhanced excitability of the hippocampal ca2 region and its contribution to seizure activity in a mouse model of temporal lobe epilepsy. Neuron.

[44] Sha L, Miller SM, Szurszewski JH (2004). Morphology and electrophysiology of neurons in dog paraventricular nucleus: in vitro study. Brain Res.

[45] St John JL, Rosene DL, Luebke JI (1997). Morphology and electrophysiology of dentate granule cells in the rhesus monkey: comparison with the rat. J Comp Neurol.

[46] Wang XM, Bey AL, Katz BM, Badea A, Kim N, David LK, Duffney LJ, Kumar S, Mague SD, Hulbert SW (2016). Altered mglur5-homer scaffolds and corticostriatal connectivity in a Shank3 complete knockout model of autism. Nat Commun.

[47] Yi F, Danko T, Botelho SC, Patzke C, Pak C, Wernig M, Sudhof TC (2016). Autism-associated Shank3 haploinsufficiency causes Ih channelopathy in human neurons. Science.

[48] Naisbitt S, Kim E, Tu JC, Xiao B, Sala C, Valtschanoff J, Weinberg RJ, Worley PF, Sheng M (1999). Shank, a novel family of postsynaptic density proteins that binds to the NMDA receptor/PSD-95/GKAP complex and cortactin. Neuron.

[49] Sala C, Vicidomini C, Bigi I, Mossa A, Verpelli C (2015). Shank synaptic scaffold proteins: keys to understanding the pathogenesis of autism and other synaptic disorders. J Neurochem.

[50] Gouder L, Vitrac A, Goubran-Botros H, Danckaert A, Tinevez JY, Andre-Leroux G, Atanasova E, Lemiere N, Biton A, Leblond CS (2019). Altered spinogenesis in iPSC-derived cortical neurons from patients with autism carrying de novo Shank3 mutations. Sci Rep.

[51] Zhao H, Tu ZC, Xu HJ, Yan S, Yan HH, Zheng YH, Yang WL, Zheng JZ, Li ZJ, Tian R (2017). Altered neurogenesis and disrupted expression of synaptic proteins in prefrontal cortex of Shank3-deficient non-human primate. Cell Res.

[52] Song TJ, Lan XY, Wei MP, Zhai FJ, Boeckers TM, Wang JN, Yuan S, Jin MY, Xie YF, Dang WW (2019). Altered behaviors and impaired synaptic function in a novel rat model with a complete Shank3 deletion. Front Cell Neurosci.

[53] Kloth AD, Badura A, Li A, Cherskov A, Connolly SG, Giovannucci A, Bangash MA, Grasselli G, Penagarikano O, Piochon C (2015). Cerebellar associative sensory learning defects in five mouse autism models. Elife.

[54] Jacot-Descombes S, Keshav NU, Dickstein DL, Wicinski B, Janssen WGM, Hiester LL, Sarfo EK, Warda T, Fam MM, Harony-Nicolas H (2020). Altered synaptic ultrastructure in the prefrontal cortex of Shank3-deficient rats. Mol Autism.

[55] Kouser M, Speed HE, Dewey CM, Reimers JM, Widman AJ, Gupta N, Liu S, Jaramillo TC, Bangash M, Xiao B (2013). Loss of predominant Shank3 isoforms results in hippocampus-dependent impairments in behavior and synaptic transmission. J Neurosci.

[56] Moessner R, Marshall CR, Sutcliffe JS, Skaug J, Pinto D, Vincent J, Zwaigenbaum L, Fernandez B, Roberts W, Szatmari P (2007). Contribution of Shank3 mutations to autism spectrum disorder. Am J Hum Genet.

[57] Jiang YH, Ehlers MD (2013). Modeling autism by shank gene mutations in mice. Neuron.

[58] Zhou Y, Sharma J, Ke Q, Landman R, Yuan J, Chen H, Hayden DS, Fisher JW, Jiang M, Menegas W (2019). Atypical behaviour and connectivity in Shank3-mutant macaques. Nature.

[59] Wang ZJ, Zhong P, Ma K, Seo JS, Yang F, Hu Z, Zhang F, Lin L, Wang J, Liu T (2020). Amelioration of autism-like social deficits by targeting histone methyltransferases ehmt1/2 in Shank3-deficient mice. Mol Psychiatry.

[60] Duffney LJ, Zhong P, Wei J, Matas E, Cheng J, Qin L, Ma K, Dietz DM, Kajiwara Y, Buxbaum JD (2015). Autism-like deficits in Shank3-deficient mice are rescued by targeting actin regulators. Cell Rep.

[61] Betancur C, Buxbaum JD (2013). Shank3 haploinsufficiency: a "common" but underdiagnosed highly penetrant monogenic cause of autism spectrum disorders. Mol Autism.

[62] Leblond CS, Nava C, Polge A, Gauthier J, Huguet G, Lumbroso S, Giuliano F, Stordeur C, Depienne C, Mouzat K (2014). Meta-analysis of shank mutations in autism spectrum disorders: a gradient of severity in cognitive impairments. PLoS Genet.

[63] Wang T, Ma J, Hogan AN, Fong S, Licon K, Tsui B, Kreisberg JF, Adams PD, Carvunis AR, Bannasch DL (2020). Quantitative translation of dog-to-human aging by conserved remodeling of the DNA methylome. Cell Syst.

[64] Courchesne E, Campbell K, Solso S (2011). Brain growth across the life span in autism: age-specific changes in anatomical pathology. Brain Res.

