# Supplementary material for: Impaired synaptic function and hyperexcitability of the pyramidal neurons in the prefrontal cortex of autism-associated Shank3 mutant dogs
Source: Mol Autism. 2024 Jan 31;15:9. doi: 10.1186/s13229-024-00587-4 (PMC10829216; doi:10.1186/s13229-024-00587-4)

**Supplementary Materials for**

**Impaired synaptic function and hyperexcitability of the pyramidal neurons in the prefrontal cortex of autism-associated *Shank3* mutant dogs**

**Supplemental Table.1: List of juvenile *Shank3* mutant dogs used in the present study.**

| Dogs | Gender | Age (Months) | Genotype |
| --- | --- | --- | --- |
| 1# | Male | 4 | -496 bp/+ |
| 2# | Male | 4 | -496 bp/+ |
| 3# | Female | 4 | -496 bp/+ |
| 4# | Male | 4 | +/+ |
| 5# | Male | 4 | +/+ |
| 6# | Male | 3.5 | +/+ |

**Supplemental Figure 1. Action potential properties of PFC pyramidal neurons in *Shank3* mutants and WT dogs.**

(**A**) Representative AP waveforms recorded in PFC pyramidal neurons from WT and *Shank3* mutant dogs.

(**B**–**H**) Summary of AP properties. AP amplitude (**B,** *p* = 0.8573), AP threshold (**C,** *p* = 0.4690), after hyperpolarization (AHP) amplitude (**D,** *p* = 0.8941), and rise time (**H,** *p* = 0.6670) show no significant changes between WT and *Shank3* mutant dogs, while AHP latency (**E,** *p* = 0.0412), half-width (**F,** *p* = 0.0490), and decay time (**G,** *p* = 0.0088) are slightly but significantly decreased in *Shank3* mutant dogs compared with WT controls. WT, n = 21 neurons from 3 dogs; mutant, n = 19 neurons from 3 dogs. Data in (**B**–**H**) were analyzed with a two-tailed, unpaired t-test. Data are presented as mean ± SEM; **p* < 0.05, ***p* < 0.01 and ****p* < 0.001.


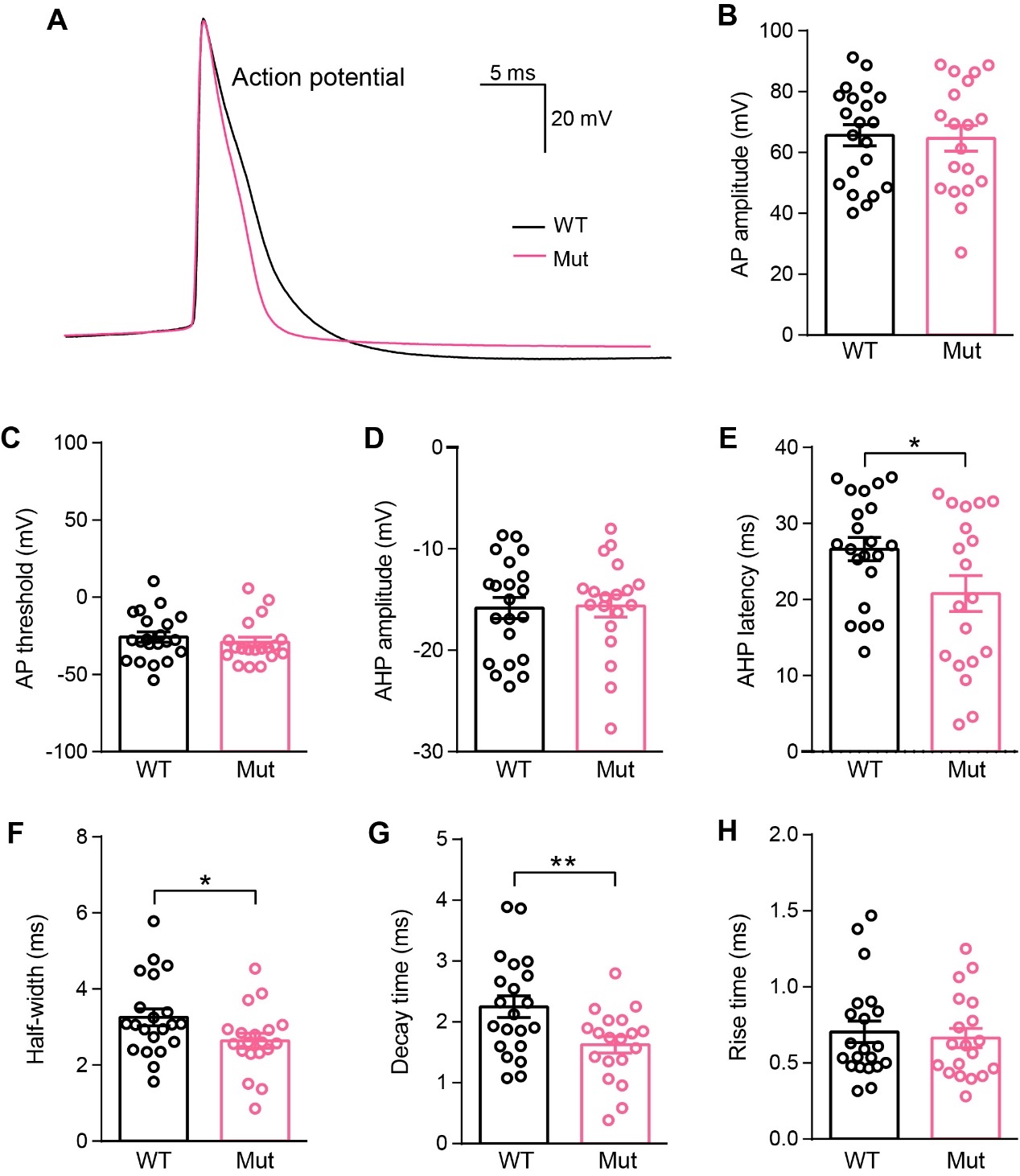


**Supplemental Figure 2：Decreased spine density in the PFC of *Shank3* mutant dogs by Golgi staining.**

(**A**) Golgi staining of PFC pyramidal neurons form WT and *Shank3* mutant dogs showing the morphology of dendritic spines.

(**B**) The spine density is lower in *Shank3* mutant dogs than the WT. n = 39 dendrites from 3 individuals of WT and *Shank3* mutant dogs aged 3–4 months old. The data points for each dog are presented as rhombus, ****p* < 0.001, two-tailed unpaired t-test. Scale bar, 10 μm.


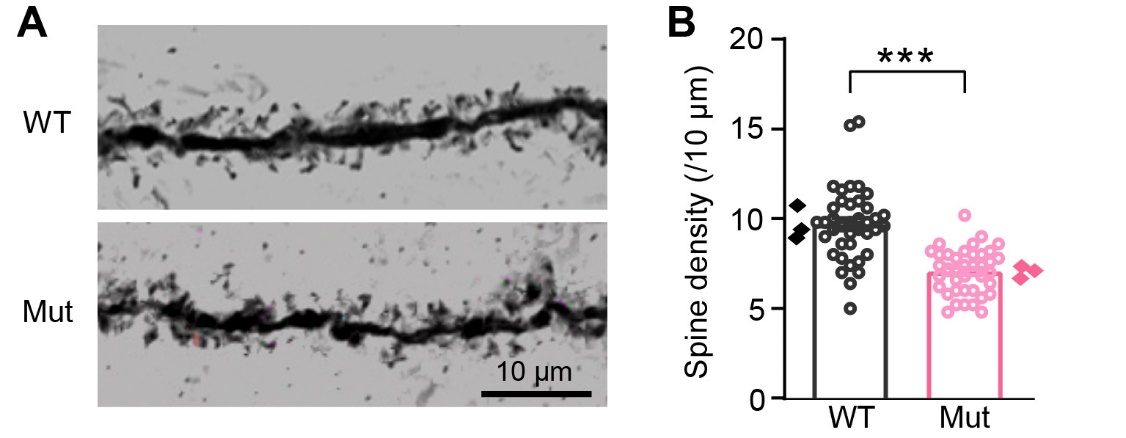

Supplement: Supplementary file 1 — Additional file 1: Supplementary table and figures. Table S1. List of juvenile Shank3 mutant dogs used in the present study. Figure S1. Action potential properties of PFC pyramidal neurons in Shank3 mutants and WT dogs. Figure S2. Decreased spine density in the PFC of Shank3 mutant dogs by Golgi staining. [file 13229_2024_587_MOESM1_ESM.docx]
